# Intravenous thrombolysis versus endovascular thrombectomy in acute basilar artery occlusion—A multicenter cohort study

**DOI:** 10.1177/17474930251344451

**Published:** 2025-05-12

**Authors:** Silja Räty, Davide Strambo, Alexandra Gomez-Exposito, João Pedro Marto, João Nuno Ramos, Stefan Krebs, Pekka Virtanen, Juhani Ritvonen, Mohamad Abdalkader, Piers Klein, Tiina Sairanen, Marek Sykora, Perttu J Lindsberg, Sven Poli, Patrik Michel, Thanh N Nguyen, Daniel Strbian

**Affiliations:** 1 Department of Neurology, Helsinki University Hospital and University of Helsinki, Helsinki, Finland; 2 Service of Neurology, Department of Clinical Neurosciences, University Hospital of Lausanne and University of Lausanne, Lausanne, Switzerland; 3 Department of Neurology & Stroke, University of Tübingen, Tübingen, Germany; 4 Hertie Institute for Clinical Brain Research, University of Tübingen, Tübingen, Germany; 5 Department of Neurology, Hospital de Egas Moniz, Centro Hospitalar Lisboa Ocidental, Lisbon, Portugal; 6 Lisbon Clinical Academic Center, NOVA Medical School, Universidade NOVA de Lisboa, Lisbon, Portugal; 7 Department of Neuroradiology, Hospital de Egas Moniz, Centro Hospitalar Lisboa Ocidental, Lisbon, Portugal; 8 Department of Neurology, St John’s Hospital Vienna, Vienna, Austria; 9 Department of Radiology, Helsinki University Hospital and University of Helsinki, Helsinki, Finland; 10 Departments of Neurology and Radiology, Boston Medical Center, Boston, MA, USA; 11 Medical Faculty, Sigmund Freud University Vienna, Vienna, Austria

**Keywords:** Basilar artery occlusion, endovascular thrombectomy, intravenous thrombolysis

## Abstract

**Background:**

Randomized controlled trials have demonstrated an improved outcome of basilar artery occlusion (BAO) with endovascular thrombectomy (EVT) compared to best medical treatment. However, a minority of the patients recruited up to 12–24 h from onset in the positive trials received intravenous thrombolysis (IVT), and a trial with a higher IVT rate did not show superiority of EVT. Thus, the efficacy and safety of EVT compared to IVT for BAO remain less clear.

**Aims:**

We aimed to compare outcomes after IVT alone to EVT with or without IVT for acute BAO.

**Methods:**

This international, observational, retrospective study included patients who received recanalization therapy for BAO at six centers between January 2010 and March 2024. The primary outcome was 3-month modified Rankin Scale (mRS) score 0–3, and secondary outcomes comprised mRS 0–2, ordinal mRS, mortality, and symptomatic intracranial hemorrhage. Outcomes after IVT versus EVT ± IVT were compared using inverse probability-weighted regression adjustment models adjusting for known predictors of outcome in BAO and baseline variables differing between the treatment groups. Interaction of the treatment group with symptom severity and onset-to-treatment time was tested.

**Results:**

Of 523 patients with BAO (median age 69, 35.2% women), 28.9% received IVT and 71.1% EVT ± IVT. The IVT-alone group had a lower baseline National Institutes of Health Stroke Scale score (median 11 vs 15) but equally extensive ischemic changes in baseline imaging. After inverse probability-weighted regression adjustment, the IVT-alone group had higher odds of mRS 0–3 (adjusted odds ratio (aOR) = 2.33 [95% confidence interval (CI) = 1.31–4.12]), mRS 0–2 (aOR = 1.93 [95% CI = 1.12–3.30]), lower median mRS (aOR = 1.81 [95% CI = 1.21–2.71]), and lower mortality (aOR = 0.53 [95% CI = 0.29–0.97]), but no difference in symptomatic intracranial hemorrhage (aOR = 0.81 [95% CI = 0.28–2.36]). No interactions for the primary outcome were found.

**Conclusion:**

In this study, patients with BAO had better outcome after IVT than EVT ± IVT independent of symptom severity and time from onset. Although the non-randomized design of the study warrants caution, the results encourage further trials comparing EVT and IVT to guide recanalization therapy in BAO patients.

**Data access statement:**

Anonymized data are available upon reasonable request to the corresponding author following the national legislation.

## Graphical abstract

**Graphical abstract:**
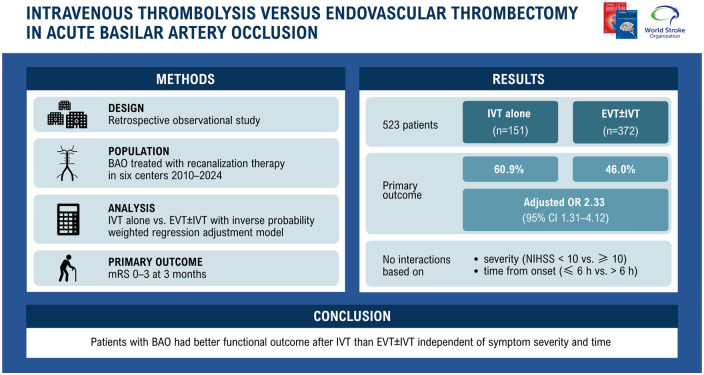

## Introduction

Basilar artery occlusion (BAO) causes about 1% of ischemic strokes but inflicts significant burden on patients and health care systems due to high mortality and morbidity.^1,2^ Because of the poor prognosis, recanalization therapies, including intravenous thrombolysis (IVT) and different endovascular approaches, have long been administered in BAO despite the lack of definitive evidence from randomized controlled trials (RCTs).

Since 2019, four RCTs comparing endovascular therapy (EVT) and best medical treatment (BMT) to BMT alone have been published^3–6^ with two of them reporting superiority of EVT in achieving favorable functional outcome.^3,4^ However, only up to one-third of patients in the BMT arms of the positive trials with a recruitment window of 12–24 h received IVT, whereas one trial with a higher IVT rate and a window of 6 h did not show a difference between the groups.^5^ Furthermore, the majority of randomized patients had moderate-to-severe symptoms. Accordingly, the European Stroke Organisation (ESO) and European Society for Minimally Invasive Neurological Therapy (ESMINT) guidelines on acute BAO recommend EVT over BMT for patients with moderate-to-severe clinical presentation. The guideline remarks that evidence is mainly derived from studies with low IVT rates, high prevalence of intracranial atherosclerosis, and primarily Asian population and acknowledges the lack of RCT-based evidence on recanalization therapy for patients with mild symptoms.^7^

Our aim was to compare outcomes of patients with acute BAO presenting with any symptom severity treated with IVT alone versus EVT with or without IVT in a large international multicenter cohort. In addition, we aimed to examine whether there is a treatment-modifying effect based on symptom severity and time from symptom onset.

## Methods

This retrospective observational multicenter cohort study consists of patients treated with recanalization therapy for acute BAO at six comprehensive stroke centers (Boston, Helsinki, Lausanne, Lisbon, Tübingen, Vienna). The selection criteria were (1) age ⩾ 18 years, (2) angiography-verified BAO, (3) recanalization therapy with IVT, EVT, or both, (4) pre-stroke mRS ⩽ 3, (5) available functional outcome data, and (6) treatment after January 2010 (applied because of use of first-generation devices and only few EVT earlier (n = 10)). The choice of recanalization therapy was left to the discretion of a clinician following local guidelines, as there were no RCTs suggesting superiority of either IVT or EVT to BMT in BAO until 2022. Patients were treated in dedicated stroke units or intensive care units.

We collected demographics, patient history, the National Institutes of Health Stroke Scale (NIHSS) score on admission, imaging findings, etiology, treatment metrics, and procedural and clinical outcomes. Ischemic changes within the posterior circulation in the baseline imaging were assessed with the posterior circulation Acute Stroke Prognosis Early CT Score (pc-ASPECTS) ranging from 0 to 10, lower values indicating more extensive early ischemia.^8^ Onset-to-treatment time was the time from last seen well to start of IVT or EVT puncture, whichever occurred first. Successful recanalization during EVT was defined as final modified treatment in cerebral infarction 2b–3.

The primary outcome was a 3-month favorable functional outcome, defined as a modified Rankin Scale (mRS) score of 0 to 3. Secondary outcomes included 3-month functional independence (mRS 0–2), distribution of the mRS (ordinal shift), and mortality, and occurrence of symptomatic intracranial hemorrhage (sICH) according to the European Cooperative Acute Stroke Study II classification, implying any type of intracranial bleeding within 7 days leading to neurological deterioration of NIHSS ⩾ 4 points or death.^9^ The functional outcome was prospectively collected at each site by investigators who were not systematically blinded to the treatment.

All centers acquired approval from an institutional review board or local ethics committee. Patient informed consent was waived due to the study’s retrospective and anonymized design. The study was reported according to the STROBE (Strengthening the Reporting of Observational Studies in Epidemiology) guideline.^10^ Anonymized data are available upon reasonable request to the corresponding author following national legislation.

### Statistical analyses

Baseline categorical variables were compared with the Pearson’s χ^2^ test and continuous variables with the Mann–Whitney *U*-test. The latter were presented as medians and interquartile ranges (IQRs).

We obtained crude odds ratios (ORs) and 95% confidence intervals (CIs) with univariable logistic regression analyses using the generalized estimating equation model considering EVT ± IVT as the reference category and IVT alone as the exposure group. An independence covariance matrix was applied to account for within-site clustering of patients. The model specifications included a logit link function and binomial distribution for the binary outcomes and a cumulative logit link function and multinomial distribution for the ordinal outcome (1-point shift toward a lower value in mRS).

Adjusted ORs were acquired with doubly robust inverse probability-weighted regression adjustment (IPWRA) models that combine propensity score-based weighting and outcome regression.^11^ In this approach, stabilized weights were applied for treatment weighting by inverse probability,^12^ and the doubly robust effect of treatment on the outcome was estimated by utilizing a logistic regression exposure model (IVT vs EVT ± IVT) and an outcome logistic regression model (3-month mRS). The models were adjusted for known predictors of outcome in BAO (age, baseline pc-ASPECTS, admission NIHSS), for baseline variables with between-group difference *p* < 0.1, and for sex and treatment period (2010–2014, 2015–2018, or 2019–2024). As outcome models, we chose the binomial logistic regression model for the binary outcomes and the multinomial logistic regression model for the ordinal outcome, using the model specifications described above. The results were expressed as ORs with 95% CIs, using EVT ± IVT as the reference level and IVT alone as the exposure group.

We tested the interaction between the treatment arm and stroke severity (NIHSS < 10) or onset-to-treatment time (⩽6 h) on the outcomes by inserting an interaction term to the adjusted analysis. Subgroup analyses according to severity and time were executed. Finally, two sensitivity analyses were performed excluding (1) patients treated before 2015 and (2) patients with onset-to-treatment time >4.5 h.

Patients with missing data were excluded from the analyses including that variable. Statistical significance was *p* < 0.05 (two-sided). Data were analyzed using the SPSS Statistics software version 28 (IBM Corp, Armonk, NY, USA).

## Results

**Figure 1.**
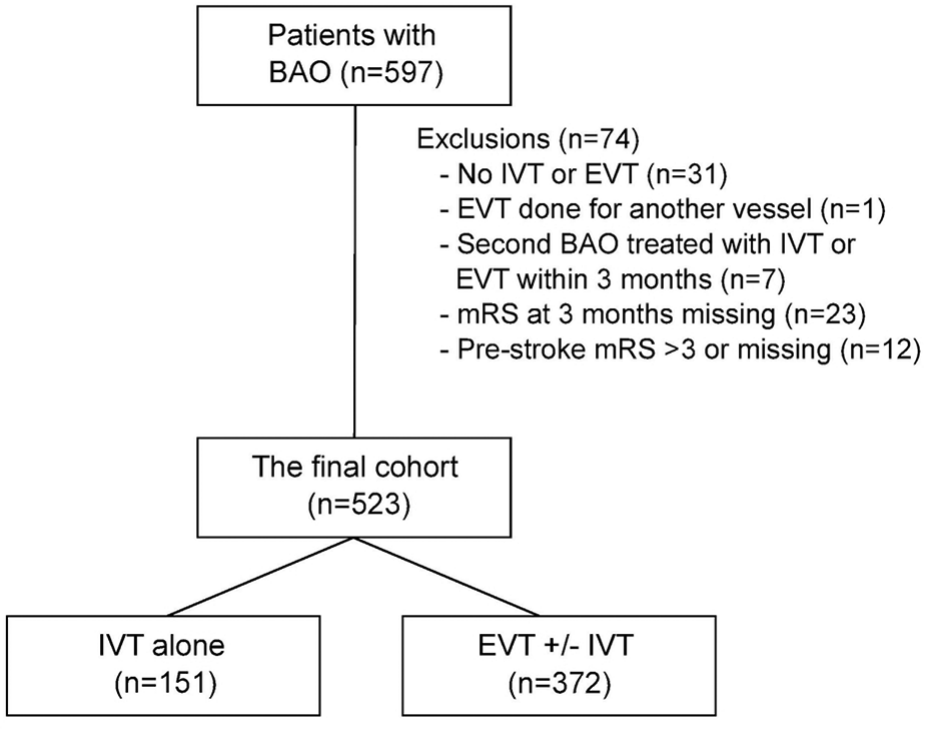
Flowchart of the study. BAO, basilar artery occlusion; IVT, intravenous thrombolysis; EVT, endovascular thrombectomy; mRS, modified Rankin Scale.

Among 597 BAO patients, 523 (87.6%) were eligible to the final cohort (Figure 1). IVT alone was administered to 151 (28.9%) and EVT ± IVT to 372 (71.1%) patients. In the EVT group, 203 (55.9%) received IVT. Median (IQR) age of the patients was 69 (60–78) years, and 35.2% were women. The EVT ± IVT group had more hypertension and higher NIHSS score on admission, but there were no other significant baseline differences (Table 1). Center-wise baseline characteristics and the number of recanalization treatments along the study years are presented in the Supplemental Material (Tables S1 and S2).

**Table 1.** Baseline characteristics of the cohort.

Baseline variables	All (*n* = 523)	IVT alone (*n* = 151)	EVT ± IVT (*n* = 372)	*p*	Missing data
Age (y), median (IQR)	69 (60-78)	71 (61-78)	69 (60-78)	0.256	0/0/0
Female sex, *n* (%)	184 (35.2)	61 (40.4)	123 (33.1)	0.130	0/0/0
Diabetes, *n* (%)	112 (21.4)	24 (15.9)	88 (23.7)	0.060	0/0/0
Hypertension, *n* (%)	355 (67.9)	92 (60.9)	263 (70.7)	0.039	0/0/0
Dyslipidemia, *n* (%)	201 (38.5)	63 (41.7)	138 (37.2)	0.372	1/0/1
Atrial fibrillation, *n* (%)	125 (23.9)	31 (20.5)	94 (25.3)	0.260	0/0/0
Coronary artery disease, *n* (%)	133 (25.4)	31 (20.5)	102 (27.4)	0.121	0/0/0
Previous ischemic stroke, *n* (%)	112 (21.5)	34 (22.5)	78 (21.0)	0.725	1/0/1
Pre-stroke mRS > 1, *n* (%)	68 (13.1)	14 (9.5)	54 (14.6)	0.149	4/3/1
NIHSS, median (IQR)	14 (7-27)	11 (5-25)	15 (7-28)	0.023	1/0/1
NIHSS < 10, *n* (%)	181 (34.7)	63 (41.7)	118 (31.8)	0.034	1/0/1
NIHSS ⩾ 20, *n* (%)	206 (39.5)	50 (33.1)	156 (42.0)	0.061	1/0/1
pc-ASPECTS, median (IQR)	10 (8-10)	10 (9-10)	10 (8-10)	0.377	9/0/9
pc-ASPECTS < 8, *n* (%)	64 (12.5)	18 (11.9)	46 (12.7)	0.884	9/0/9
BAO ⩾ 2 segments, *n* (%)	205 (42.9)	54 (38.0)	151 (44.9)	0.181	45/9/36
Large-artery atherosclerosis, *n* (%)	178 (35.5)	57 (37.7)	121 (34.5)	0.542	21/0/21
Cardioembolism, *n* (%)	183 (36.5)	57 (37.7)	126 (35.9)	0.762	21/0/21
Other determined etiology, *n* (%)	40 (8.0)	8 (5.3)	32 (9.1)	0.156	21/0/21
Undetermined etiology, *n* (%)	107 (21.3)	31 (20.5)	76 (21.7)	0.813	21/0/21
IVT, *n* (%)	359 (68.6)	151 (100)	208 (55.9)	< 0.001	0/0/0
Onset to treatment (h), median (IQR)	3.8 (2.1-9.3)	3.1 (2.0-9.7)	4.0 (2.2-9.3)	0.289	24/2/22
Onset to treatment 0-6 h, *n* (%)	325 (64.9)	104 (69.8)	221 (62.8)	0.152	22/2/20
Onset to IVT (h), median (IQR)	2.9 (1.9-5.9)	3.1 (2.0-9.7)	2.7 (1.8-5.7)	0.040	174/2/172
Onset to puncture (h), median (IQR)	.	.	5.6 (3.4-9.8)	.	26
First-pass technique, *n* (%)	.	.	.	.	2
Stent retriever	.	.	116 (31.4)	.	.
Aspiration	.	.	132 (35.7)	.	.
Combined stent retriever + aspiration	.	.	88 (23.8)	.	.
Other	.	.	23 (6.2)	.	.
No access to clot	.	.	11 (3.0)	.	.
No. of passes, median (IQR)	.	.	2 (1-3)	.	4
Stenting, *n* (%)	.	.	46 (12.4)	.	1
Successful recanalization, *n* (%)	.	.	300 (81.5)	.	4
Antithrombotic medication ⩽ 24 h	.	.	185 (50.1)	.	3

IVT, intravenous thrombolysis; EVT, endovascular thrombectomy; mRS, modified Rankin Scale; NIHSS, National Institutes of Health Stroke Scale; pc-ASPECTS, posterior circulation Acute Stroke Prognosis Early CT Score; BAO, basilar artery occlusion.

Regarding EVT, the most common first-pass technique was aspiration (35.7%), followed by a stent retriever use (31.4%) (Table 1). Successful recanalization occurred in 81.5%. Add-on antithrombotic medication within 24 hours was administered to 50.1%: aspirin monotherapy (11.9%), therapeutic-dose low-molecular heparin (11.4%), unfractionated heparin (7.0%), glycoprotein IIb/IIIa inhibitors (5.4%), dual antiplatelet (3.8%), or combination (10.3%). Early reocclusion (⩽ 24 h after successful recanalization) was experienced by 6.8%, and procedure-related complications included access complication (2.7%), dissection (2.2%), distal embolization (2.2%), perforation (1.9%), and treated vasospasm (1.4%).

The primary outcome was achieved by 60.9% of the IVT-alone group and 46.0% of the EVT ± IVT group. According to the IPWRA analysis, the patients receiving IVT alone had higher odds of favorable functional outcome (adjusted odds ratio (aOR) = 2.33 [95% CI = 1.31–4.12]) (Table 2). Of the secondary outcomes, functional independence was more frequent (46.4% vs 34.4%; aOR = 1.93 [95% CI = 1.12–3.30]) and mRS distribution better after IVT (median = 3 [IQR = 2–6] vs 4 [2–6]; aOR = 1.81 [95% CI = 1.21–2.71]). Mortality was 29.8% in the IVT-alone group and 40.3% in the EVT ± IVT group (aOR = 0.53 [95% CI = 0.29–0.97]) and the sICH rate 6.0% and 5.2%, respectively (aOR = 0.81 [95% CI = 0.28–2.36]).

**Table 2.** Univariable and inverse probability-weighted regression adjustment comparison of outcomes in the treatment arms.

Outcome	IVT alone (*n* = 151)	EVT ± IVT (*n* = 372)	Missing	Unadj. OR (95% CI)^a^	*p*	IPWRA OR (95% CI)^b^	*p*
3-month mRS 0–3, *n* (%)	92 (60.9)	171 (46.0)	0/0	1.83 (1.25-2.69)	0.002	2.33 (1.31-4.12)	0.004
3-month mRS 0–2, *n* (%)	70 (46.4)	128 (34.4)	0/0	1.65 (1.12-2.42)	0.011	1.93 (1.12-3.30)	0.017
3-month mRS, median (IQR)	3 (2-6)	4 (2-6)	0/0	1.60 (1.14-2.23)^c^	0.007	1.81 (1.21-2.71)^c^	0.004
Mortality, *n* (%)	45 (29.8)	150 (40.3)	0/0	0.63 (0.42-0.94)	0.025	0.53 (0.29-0.97)	0.027
sICH, *n* (%)	9 (6.0)	19 (5.2)	1/7	1.16 (0.51-2.60)	0.727	0.81 (0.28-2.36)	0.695

a Univariable generalized estimating equation analysis.

b Inverse probability-weighted regression adjustment (IPWRA) model, including treatment group (IVT only vs EVT ± IVT), age, sex, diabetes, hypertension, the National Institutes of Health Stroke Scale, posterior circulation Acute Stroke Prognosis Early CT Score < 8, and treatment period (2010–2014, 2015–2018, or 2019–2024).

c Common OR.

IVT, intravenous thrombolysis; EVT, endovascular thrombectomy; mRS, modified Rankin Scale; sICH, symptomatic intracranial hemorrhage.

**Figure 2.**
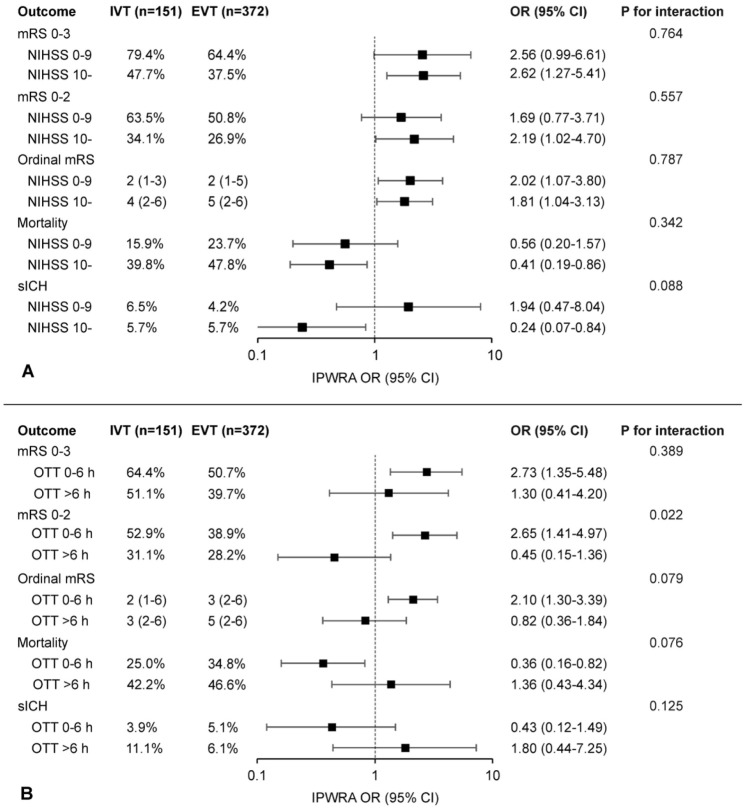
Outcomes according to the inverse probability-weighted regression adjustment model stratified by baseline symptom severity and time from onset. Forest plot of adjusted ORs and 95% CIs from the doubly robust inverse probability-weighted regression adjustment (IPWRA) analysis for comparison of intravenous thrombolysis (IVT) and endovascular thrombectomy (EVT) ± IVT (reference category) in basilar artery occlusion stratified by the National Institutes of Health Stroke Scale (NIHSS) 0–9 vs  > 10 (panel A) and onset-to-treatment time (OTT) 0-6 vs >6 h (panel B). Models included treatment group (IVT only vs EVT ± IVT), age, sex, diabetes, hypertension, NIHSS, posterior circulation Acute Stroke Prognosis Early CT Score (pc-ASPECTS < 8), and treatment period (2010–2014, 2015–2018, or 2019–2024), except for the model for symptomatic intracranial hemorrhage (sICH) in the subgroup of NIHSS 0–9 that did not converge with pc-ASPECTS. mRS, modified Rankin Scale.

There was no interaction between the treatment and NIHSS cutoff of 10 (*p* = 0.764) or onset-to-treatment time 6 h (*p* = 0.457) for the primary outcome. Considering the secondary outcomes, we detected an interaction between onset-to-treatment time and the treatment arm for mRS 0–2 (*p* = 0.022) (Figure 2). The results of the primary outcome did not change in either of the sensitivity analysis, but there was no longer a difference in mRS 0–2 or mortality after excluding patients treated before 2015 (Supplemental Material, Tables S3 and S4).

## Discussion

In this multicenter observational cohort of 519 patients with acute BAO, patients treated with IVT alone had higher odds of favorable functional outcome at 3 months compared to patients treated with EVT ± IVT. The result was congruent for all functional outcomes, and there was no interaction with baseline symptom severity or time from onset on the primary outcome.

Although two of the four RCTs showed superiority of EVT over BMT for BAO patients with moderate-to-severe symptoms, the BMT consisted mostly of antithrombotic medication and not IVT.^3,4^ The lower use of IVT is likely related to the longer time windows (up to 12–24 h) in which patients were enrolled. Although one trial found no interaction according to IVT administration, the benefit of EVT was not as clear in the IVT-treated subgroup.^3^ In the RCT with an approximately 80% IVT rate, the outcome of BMT-treated patients was better compared to the other RCTs, and the trial showed no benefit of EVT.^5^

Observational studies on BAO have revealed similar results: EVT has been superior to BMT in cohorts with a low IVT rate,^13,14^ whereas studies comparing EVT and IVT have indicated opposite.^2,7,15^ The multicenter Basilar Artery International Cooperation Study (BASICS) registry found no difference between IVT and intra-arterial therapy for patients with severe symptoms and detected better functional outcome after IVT when symptoms were mild to moderate.^2^ However, IVT included subsequent intra-arterial thrombolysis in one-third of cases and thrombectomy techniques have since evolved. In a more recent cohort from one of the present study centers, the IVT-alone group had better functional outcome than BAO patients treated with EVT ± IVT.^15^ Compared to the previous study, it was our intention to specifically enrich the EVT cohort to include patients treated with modern devices and from multiple centers, as the thrombolysis molecule is the same, but the procedure-related factors may differ between centers. Consequently, the current cohort included a threefold number of patients treated with EVT from six centers with almost equal functional outcome to the previous study, indicating that the result was not based on worse procedural success in a single center, nor on selecting only patients with poor prognosis for EVT.

The outcome of the EVT group in our study is in line with the RCTs, but the patients receiving IVT alone did better than expected.^3–6^ The IVT group had lower baseline NIHSS, implying that clinicians tended to choose EVT for patients with more severe symptoms, even though one-third of the IVT-treated patients had NIHSS ⩾ 20. Symptom severity was adjusted for in the IPWRA analysis; however, it is possible that for some patients EVT was either performed or withheld due to symptom progression or improvement after IVT. In previous studies, including an individual-patient data meta-analysis of the RCTs, patients with moderate-to-severe symptoms benefited from EVT over BMT, whereas no clear treatment effect for patients with NIHSS < 10 was shown.^16^ Similarly, in observational studies, the comparison of EVT and BMT has been mostly neutral in mild symptoms^14,17^ or even favored IVT alone.^2,18^

Over 30% of the study patients, independent of the group, were treated later than 6 h after the onset. The guideline-based time window during the study was mostly 4.5 h for IVT and 6 h for EVT,^19–21^ even though windows up to 24 h for anterior circulation large-vessel occlusion have been proven effective and safe.^22,23^ Considering BAO, one RCT included patients within 24 hours,^4^ whereas some observational studies have reported even longer treatment windows.^15,24,25^ Indeed, the extent of baseline ischemic changes has been demonstrated to be a more accurate predictor of outcome than time, suggesting that BAO patients can benefit from recanalization in longer time windows in case of discrepancy between symptoms and established infarction.^25^ This study found no interaction of time and choice of treatment for favorable functional outcome. However, patients achieved more often functional independence with IVT alone within 6 h, whereas there was no difference to EVT after that, and a trend for concordant findings was detected regarding median mRS and mortality.

The reason for not observing similar outcome improvement after EVT compared to IVT in BAO as in anterior circulation large-vessel occlusion is unclear.^26^ One explanation may stem from the higher recanalization rate of IVT in posterior compared to anterior circulation occlusions, when adjusted for thrombus length.^27,28^ Conversely, distinct features of the collateral network and blood flow of the posterior circulation could predispose patients to clinical deterioration in case of thrombus migration, distal embolization, or rethrombosis during EVT.^29^ We observed early reocclusion in 6.8% after EVT, which is equal^30,31^ or higher^32^ compared to observational reports comprising mostly anterior circulation occlusions. Other procedural complications were rare and in line with the RCTs.^3–6^

The study strengths include its sample size, multicenter design, and robust statistical methods to minimize confounding. However, it also possesses limitations. First, the non-randomized design resulted in some baseline imbalances between the treatment groups. EVT was increasingly used during the later study years and more often chosen for patients with severe symptoms. Although these differences were considered in the IPWRA model, a risk of residual confounding exists. Second, the number of affected BAO segments was missing from 8.7%, and no data on the basilar artery patency at 24 h were available. Third, imaging analysis was not centralized but performed locally in the participating centers. Fourth, the study population is collected from Europe and North America, hindering direct comparisons to the RCTs with predominantly Asian populations. For instance, large-artery atherosclerosis was less prevalent in our cohort compared to the RCTs.^3,4,6^ Finally, most of the patients in the IVT group were from one center (Helsinki) that has an established protocol for the use of IVT for BAO in time windows exceeding 4.5 h.^24,25^ We acknowledge that this is not a universal strategy, albeit used in some centers. Furthermore, IVT up to 24 h was recently endorsed by an expert consensus in the ESO-ESMINT guideline, encouraging this approach worldwide and especially in centers without immediate access to EVT.^7^

## Conclusion

This study observed more favorable outcome after IVT compared to EVT with or without IVT for acute BAO. Although the results were most consistent within 6 h of onset, there was no interaction for favorable functional outcome based on the time window, and symptom severity did not modify the results. Considering the limitations of an observational design, our results do not question the findings of the RCTs. Instead, they highlight the role of IVT as a pivotal part of the recanalization treatment for BAO and suggest that IVT can be considered in longer time windows than currently applied in most centers. Further trials comparing IVT and EVT up to 24 h and including also patients with mild symptoms are needed to guide the optimal recanalization strategy in BAO.

## Supplemental Material

sj-pdf-1-wso-10.1177_17474930251344451 – Supplemental material for Intravenous thrombolysis versus endovascular thrombectomy in acute basilar artery occlusion—A multicenter cohort study Supplemental material, sj-pdf-1-wso-10.1177_17474930251344451 for Intravenous thrombolysis versus endovascular thrombectomy in acute basilar artery occlusion—A multicenter cohort study by Silja Räty, Davide Strambo, Alexandra Gomez-Exposito, João Pedro Marto, João Nuno Ramos, Stefan Krebs, Pekka Virtanen, Juhani Ritvonen, Mohamad Abdalkader, Piers Klein, Tiina Sairanen, Marek Sykora, Perttu J Lindsberg, Sven Poli, Patrik Michel, Thanh N Nguyen and Daniel Strbian in International Journal of Stroke
