# Supplementary material for: Intravenous thrombolysis versus endovascular thrombectomy in acute basilar artery occlusion—A multicenter cohort study
Source: Int J Stroke. 2025 May 12;20(9):1114–22. doi: 10.1177/17474930251344451 (PMC12521756; doi:10.1177/17474930251344451)
Supplement: sj-pdf-1-wso-10.1177_17474930251344451 – Supplemental material for Intravenous thrombolysis versus endovascular thrombectomy in acute basilar artery occlusion—A multicenter cohort study [file sj-pdf-1-wso-10.1177_17474930251344451.pdf]

SUPPLEMENTAL MATERIAL

Intravenous Thrombolysis Versus Endovascular Thrombectomy in Acute Basilar Artery Occlusion – A Multicenter Cohort Study

Silja Räty, Davide Strambo, Alexandra Gomez-Exposito, João Pedro Marto, João Nuno Ramos, Stefan Krebs, Pekka Virtanen, Juhani Ritvonen, Mohamad Abdalkader, Piers Klein, Tiina Sairanen, Marek Sykora, Perttu J. Lindsberg, Sven Poli, Patrik Michel, Thanh N. Nguyen, Daniel Strbian

Table S1. Baseline characteristics according to center

Variables	Boston (n=21)	Helsinki (n=242)	Lausanne (n=97)	Lisbon (n=41)	Tübingen (n=81)	Vienna (n=41)
Years	2015-2021	2010-2022	2010-2022	2016-2022	2015-2023	2014-2024
Age (y), median (IQR)	57 (53-70)	69 (60-77)	70 (61-80)	69 (63-80)	73 (64-80)	69 (61-81)
Female sex, n (%)	7 (33)	82 (34)	30 (31)	17 (41)	31 (38)	17 (41)
NIHSS, median (IQR)	20 (8-27)	13 (6-27)	14 (9-32)	24 (13-33)	13 (6-25)[a]	13 (6-26)
pc-ASPECTS, median (IQR)	9 (7-10)	10 (8-10)	10 (8-10)	10 (9-10)[b]	10 (9-10)[a]	9 (8-10)
IVT alone, n (%)	0	118 (49)	16 (16)	7 (17)	6 (7)	6 (15)
OTT 0-6 h, n (%)	8 (40)[a]	151 (62)	65 (67)	21 (51)	60 (80)[c]	20 (77)[d]

[a]Missing n=1. [b]Missing n=8. [c]Missing n=6. [d]Missing n=15. IQR, interquartile range; NIHSS, National Institutes of Health Stroke Scale; pc-ASPECTS, posterior circulation Acute Stroke Prognosis Early CT Score; IVT, intravenous thrombolysis; EVT, endovascular thrombectomy; OTT, onset-to-treatment time.

Table S2. Recanalization treatment according to study years

Treatment years	All (n=523)	IVT alone (n=151)	EVT+/-IVT (n=372)
2010-2014, n (%)	109 (20.8)	71 (47.0)	38 (10.2)
2015-2018, n (%)	191 (36.5)	47 (31.1)	144 (38.7)
2019-2024, n (%)	223 (42.6)	33 (21.9)	190 (51.1)

IVT, intravenous thrombolysis; EVT, endovascular thrombectomy

Table S3. Outcomes according to treatment (IVT alone versus EVT with or without IVT) in a subgroup of patients treated between 2015 and 2024

Outcomes	IVT alone (n=80)	EVT+/-IVT (n=334)	Missing	IPWRA OR (95% CI)*	*P*
3-month mRS 0-3, n (%)	57 (71.3)	158 (47.3)	0/0	2.16 (1.16-4.00)	0.015
3-month mRS 0-2, n (%)	44 (55.0)	120 (35.9)	0/0	1.71 (0.94-3.08)	0.077
3-month mRS, median (IQR)	2 (1-4)	4 (2-6)	0/0	1.62 (1.03-2.54)†	0.036
Mortality, n (%)	17 (21.3)	133 (39.8)	0/0	0.54 (0.27-1.07)	0.077
sICH, n (%)	3 (3.8)	15 (4.6)	1/5	0.52 (0.13-2.09)	0.357

*Inverse probability weighted regression adjustment (IPWRA) model, including treatment group (IVT only vs EVT+/-IVT), age, sex, diabetes, hypertension, National Institutes of Health Stroke Scale, posterior circulation Acute Stroke Prognosis Early CT Score (pc-ASPECTS<8), and treatment period (2015-2018 or 2019-2024). †Common odds ratio (OR). IVT, intravenous thrombolysis; EVT, endovascular thrombectomy; mRS, modified Rankin Scale; IQR, interquartile range; sICH, symptomatic intracranial hemorrhage.

Table S4. Outcomes according to treatment (IVT alone versus EVT with or without IVT) in a subgroup of patients treated within 4.5 hours of symptom onset

**Outcomes**	**IVT alone (n=95)**	**EVT+/-IVT (n=189)**	**Missing**	**IPWRA OR (95% CI)***	***P***
3-month mRS 0-3, n (%)	62 (65.3)	99 (52.4)	0/0	2.23 (1.02-4.89)	0.046
3-month mRS 0-2, n (%)	53 (55.8)	75 (39.7)	0/0	2.66 (1.33-5.31)	0.006
3-month mRS, median (IQR)	2 (1-5)	3 (2-6)	0/0	1.94 (1.16-3.25)†	0.012
Mortality, n (%)	23 (24.2)	64 (33.9)	0/0	0.41 (0.17-0.99)	0.047
sICH, n (%)	4 (4.3)	6 (3.3)	1/5	1.20 (0.33-4.35)	0.782

*Inverse probability weighted regression adjustment (IPWRA) model, including treatment group (IVT only vs EVT+/-IVT), age, sex, diabetes, hypertension, National Institutes of Health Stroke Scale, posterior circulation Acute Stroke Prognosis Early CT Score (pc-ASPECTS<8), and treatment period (2010-2014, 2015-2018, or 2019-2022). †Common odds ratio (OR). IVT, intravenous thrombolysis; EVT, endovascular thrombectomy; mRS, modified Rankin Scale; IQR, interquartile range; sICH, symptomatic intracranial hemorrhage.
